# Why do graduates choose to work in a less attractive specialty? A cross-sectional study on the role of personal values and expectations

**DOI:** 10.1186/s12960-020-00474-y

**Published:** 2020-05-04

**Authors:** Van Anh Thi Nguyen, Karen D. Könings, E. Pamela Wright, Giang Bao Kim, Hoat Ngoc Luu, Albert J. J. A. Scherpbier, Jeroen J. G. van Merriënboer

**Affiliations:** 1 Department of Medical Education and Skills Laboratory, Hanoi Medical University, Room 504, B4 Building, 1 Ton That Tung Street, Dongda, Hanoi, 10000 Vietnam; 2 School of Health Professions Education (SHE), Faculty of Health, Medicine and Life Sciences, Maastricht University, P.O. Box 616, 6200 MD Maastricht, the Netherlands; 3 Guelph International Health Consulting, Frederik Hendrikstraat 18, 1052 HT Amsterdam, the Netherlands; 4 Center of Student Assessment and Quality Assurance, Hanoi Medical University, 1 Ton That Tung Street, Dongda, Hanoi, Vietnam; 5 Biostatistics and Medical Informatics Department, Institute for Preventive Medicine and Public Health, Hanoi Medical University, 1 Ton That Tung Street, Dongda, Hanoi, Vietnam

**Keywords:** Primary health care, Preventive medicine, Role in life, Satisfaction with job, Recruitment, Retention, Career decision

## Abstract

**Background:**

Primary health care (PHC), of which preventive medicine (PM) is a subspecialty, will have to cope with a deficiency of staff in the future, which makes the retention of graduates urgent. This study was conducted in Vietnam, where PM is an undergraduate degree in parallel to medical training. It aims to identify facilitating and hindering factors that impact recruitment and retention of PM graduates in the specialty.

**Methods:**

A cross-sectional study enrolled 167 graduates who qualified as PM doctors from a Vietnamese medical school, between 2012 and 2018. Data were collected via an online questionnaire that asked participants about their motivation and continuation in PM, the major life roles that they were playing, and their satisfaction with their job. Multiple regression analyses were used to identify which life roles and motivational factors were related to the decision to take a PM position and to stay in the specialty, as well as how these factors held for subgroups of graduates (men, women, graduates who studied PM as their first or second study choice).

**Results:**

Half of the PM graduates actually worked in PM, and only one fourth of them expressed the intention to stay in the field. Three years after qualification, many graduates had not yet decided whether to pursue a career in PM. Satisfaction with opportunities for continuous education was rated as highly motivating for graduates to choose and to stay in PM. Responsibility for taking care of parents motivated male graduates to choose PM, while good citizenship and serving the community was associated with the retention of graduates for whom PM was their first choice.

**Conclusions:**

The findings demonstrate the importance of social context and personal factors in developing primary care workforce policy. Providing opportunities for continued education and enhancing the attractiveness of PM as an appropriate specialty to doctors who are more attached to family and the community could be solutions to maintaining the workforce in PM. The implications could be useful for other less popular specialties that also struggle with recruiting and retaining staff.

## Background

Despite many efforts to increase the number of physicians serving in primary health care (PHC) specialties, there is still disparity in the distribution of health care staff between hospital and community settings [1, 2]. The need for physicians serving in PHC areas is, however, expected to grow, especially in middle- and low-income countries (MLICs) [2–4]. In general, medical students’ and graduates’ career preference for primary care specialties has decreased over time [5–7]. For example, in the United Kingdom, more than 70% of recently qualified doctors expressed preference for a hospital specialty, while 25% specified general practice, and only 1% chose public or community health [8]. There are reasons to expect that the problem will be the same or even larger in MLICs, where fewer medical students choose a PHC career [9, 10]; if they do choose PHC, it is often as a “second choice” specialty [11]. Furthermore, PHC doctors tend to leave their jobs in the community and seek a hospital position after completing post-graduate specialty training [2, 12–14]. This suggests that scarce resources of students, medical schools, and society may be wasted and result in the diminishing number of physicians in the PHC field.

Although there are several reports on career choices of health care workers who graduated as medical doctors in rural and remote areas [15–17], little is known about the factors related to motivation of those who studied and graduated from the less popular specialties, such as PHC. In Vietnam, preventive medicine (PM) is a subspecialty of PHC; it constitutes an undergraduate curriculum program that trains specialized staff to work in PM centers and in the community and leads to a Doctor of Preventive Medicine degree. However, our recent study [10] showed that one third of the students who began their study in PM had little knowledge about the specialty and that it was a “second choice” when they failed to qualify for medical school. As a consequence, these “second-choice” students expressed regret and wanted to pursue a “higher prestige” clinical position in the hospital. The tendency of moving away from the community of PHC workers has been reported both in Vietnam [12, 14] and in other countries [13, 15, 17]. The main aim of the current study was to understand the time period at which PHC graduates, specifically graduates in PM, decide on their choice of job and the facilitating and hindering factors that affect their retention in the specialty.

Literature on clinical career paths suggests that it could take at least 3 years after graduation for most medical doctors to make their final decision on career choice [18, 19]. We do not know yet if this period of 3 years would be applicable to PM doctors and, if so, what happens during that time. Addressing these questions would help provide better counseling for PM students and young doctors, especially to those for whom PM was not the first preference career, so that there is greater continuity in their medical career planning.

Several demotivating factors that might impact the decision to maintain or change specialty among PHC doctors have been mentioned in the context of MLICs [15–17]: low salaries; fewer opportunities for career development; inadequate management, supervision, and training; poor infrastructure and resources; and difficult working conditions. Incentives other than finances play a critical role in increasing PHC staff motivation, including recognition or appreciation by employer, colleagues, and the community; having a stable job and income; and access to training [15, 17]. Intrinsic factors, such as love for the work or “being useful to society and taking care of people,” have also been noted [16]. In contrast, factors in high-income countries seem to be more often related to individual and family issues [19, 20]. For instance, domestic circumstances, perceived lack of collegial support, social isolation from family and friends, and lack of post-graduate training were listed as demotivating factors for staff working in remote areas in Canada and Australia [20]. In European countries, female graduates had greater preference for a community specialty and domestic circumstances were more relevant for female than for male doctors; they were also more important for general practice physicians than for hospital doctors [19]. These findings might be related to a community specialty’s greater compatibility with family life compared to a hospital specialty.

**Fig. 1:**
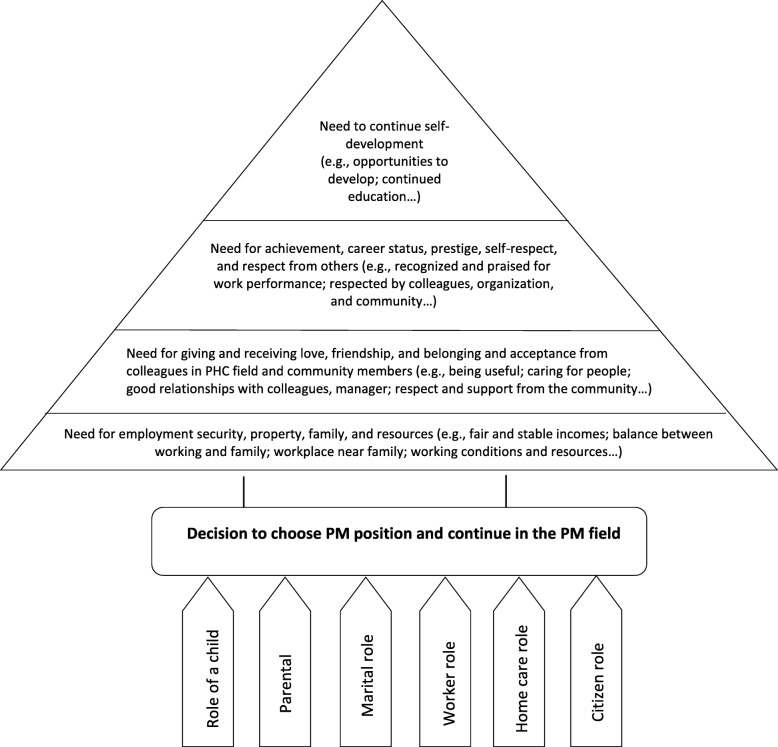
Needs of preventive medicine graduates

Theoretically, motives for important decisions in life can be understood using Maslow’s motivation theory [21]. According to Maslow, an individual cannot be fulfilled in life unless all five fundamental elements (i.e., physiological needs and needs for safety, love and belonging, esteem, and self-actualization) are met, starting from basic physiological needs and working toward the highest level, self-actualization. Since work plays an important role in the quality of life, Maslow’s theory can be applied to explain work-related needs in an individual’s career. Furthermore, the needs of a person could change during their lifetime in tandem with their age and personal and professional experiences and responsibilities. According to Super’s theory of life role in career development [22], at a certain stage of life, people can simultaneously play multiple roles, as a child, student, citizen, worker, and homemaker; increasing the number of roles in one’s life may mean less commitment to other roles. This theory has been applied to human resource development in a diverse range of contexts [23]. Applying these theories to define PM graduates’ working needs in relation to their roles at the time they make their career decision may give clues to understanding their motivation and expectations for their career pathway. For example, female doctors are expected to fulfill the simultaneous roles of family caretaker and physician; therefore, the need to be settled and able to take care of family might impact their consideration of working in the community. Another example would be if graduates have to support their wider family, which might lead to a choice to work near their parents in their hometown or encourage them to find a position in a hospital based on better income and access to health care for family members. The application of Maslow’s and Super’s theories to understand graduates’ decision to choose and continue with PM is presented in Fig. 1.

There are gaps in our knowledge of the possible impacts of need fulfillment at particular life stages on graduates’ motivation to choose a job in PM (a subspecialty of PHC) and continue in the specialty. The objective of this study was to identify the reasons related to life roles and motivational factors that lead to PM graduates choosing a PM position and staying in the field.

## Methods

### Settings and participants

Hanoi Medical University (HMU) has graduated eight cohorts of PM students since the program started in 2006. Students are recruited as high school graduates for a 6-year track: the first 2 years in the curriculum focus on basic medical knowledge and skills, the next two on clinical clerkships, and the last two on knowledge and skills related to the PM specialty. In 2013, a 4-year program started recruiting students already holding a Bachelor of Science degree in nursing, public health, and medical technology; it covers only the last 4 years of the 6-year curriculum. This study was conducted among 288 PM graduates from the 6-year track between 2012 and 2018. We did not include the 4-year track graduates because of limited resources and because they may differ significantly in background, prior experience in health care, and motivations to study PM.

### Procedure

**Fig. 2:**
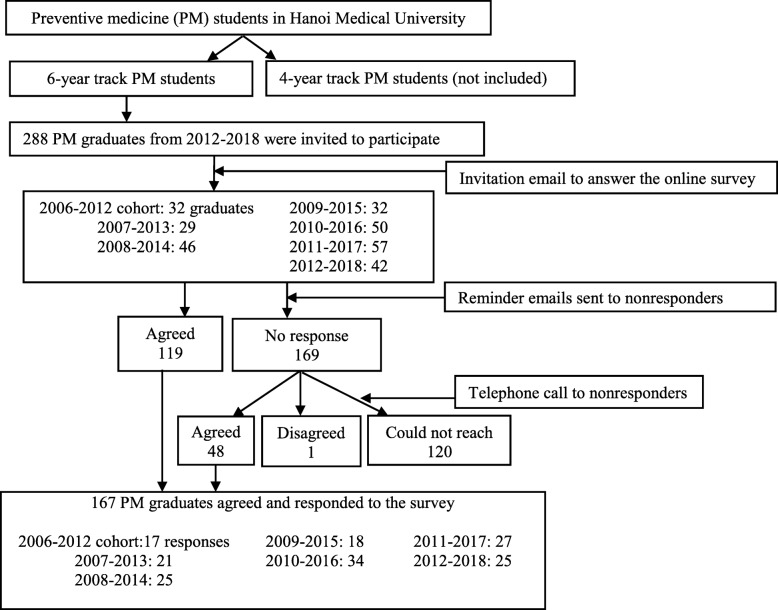
Flowchart of participant recruitment process

PM graduates’ contact information was collected through administrators and alumni networks, using a snowball technique. Graduates were invited to participate by an email containing a brief introduction of the study and a link to the online survey (Google form). Two reminder emails were sent to those who did not respond after 2 and 4 weeks, and finally, a telephone interview was conducted to get the highest rate of response and to clarify unclear answers. The recruitment process is summarized in Fig. 2. Ethical approval for the study was given by the Institutional Review Board of HMU (Decision No 174/HMU-IRB).

### Materials

A four-part questionnaire with 89 items (Additional file 1) was developed based on previous relevant scales [24–26]. Part A (eight items) investigated demographic information of participants, their current work site, and level of the health organization in the state health system where they were working. Part B (13 items) measured graduate’s personal decision of choosing a PM position, the strength of their attachment to the PM specialty, and their desire to work in PM or intention to leave the field if provided the opportunity. Part C (35 items) was developed based on Maslow’s motivation theory [21] to measure PM graduates’ job satisfaction (in seven dimensions: job and working conditions, management, coworkers, promotion, pay, contingent rewards, and continued education). Relevant items were selected from existing job satisfaction scales [24, 25], and new items were formulated specific to the PM specialty*.* Part D (33 items) was developed based on Super’s theory of life role [22] to assess six major roles in a PM graduate’s life (role of a child, parent, marital partner, worker, homemaker, and citizen). Relevant items were selected from existing life role scale [26], and two new subscales were formulated: one corresponding to the role of a child (to measure the commitment to staying close to and taking care of parents, an important task of a child in Vietnamese culture) and one for the role of a citizen (to measure the commitment to serving the community, reflecting a presumed characteristic of a PM doctor).

For all items in parts B, C, and D, participants were asked to indicate to what extent they agreed or disagreed with the statement on a 5-point Likert scale (1 = strongly disagree, 2 = disagree, 3 = neither agree nor disagree, 4 = agree, 5 = strongly agree). A pilot survey was conducted with five PM graduates to obtain feedback on the form, which was then revised to improve clarity.

Exploratory factor analysis was used to identify factors within the subscales related to personal decision-making on job choice and continuing with PM. The Cronbach’s alpha was calculated for each subscale and was improved by removing items that reduced the internal consistency of that subscale. Table 1 presents the content and internal consistency reliabilities of the 15 subscales.

**Table 1:** Study instrument and internal consistency of the subscales after conducting exploratory factor analysis and deleting items

	Subscales	Example item	Cronbach’s *α*
Demographic information (part A, 9 items)	Personal information Current working place Time of making decision	Gender, marital status, number of children… Place of work, level of organization Time of making decision of specialty choice	NA
Job choice and staying in preventive medicine (PM) (part B, 4 items)	Choosing PM job	I accepted this job because it relates the most to PM.	.67
	Continuing in PM	I wish to work in the PM field if I have the opportunity.	NA
Motivational factors (part C, 35 items)	Happy with job and working conditions	I am satisfied with my job in terms of working conditions.	.85
	Happy with manager	My manager always stands behind the workers.	.94
	Happy with colleagues	I am satisfied with the people I speak and work with.	.93
	Happy with promotion	I am satisfied with the opportunity of being promoted at this job.	.87
	Happy with pay	My salary is good when it is compared with the wage of other physicians who work at similar positions in other specialties.	.91
	Happy with reward	I feel respected and supported while working with people in the community.	.72
	Happy with continued education	My opportunities for continued education are appropriate when compared to physicians who work in other specialties.	.90
Multiple roles in life (part D, 28 items)	Child role	I tried to find a job with which I can afford to support my parents and family in my hometown.	.62
	Parental role	I tried to find a job which allowed me to have time to take care of my children.	.60
	Marital role	I tried to find a job which allowed me to have time for my spouse.	.76
	Worker role	I tried to find a job that was interesting and exciting to me.	.74
	Homecare role	I tried to find a job which allowed me to have time to manage and care for my home.	.72
	Citizen role	I tried to find a job in which I can contribute to the community where I originate from.	.58

Note: On the parental and marital role subscales, participants who were not married or had no children indicated “Not applicable” for all items, which caused missing values when calculating the mean score of the subscales. To prevent violations of the reliability of a regression model cause by a small sample size, we did not use these two subscales in further analyses but two equivalent variables: number of children (1 = no child, 2 = have one child, 3 = have two children, 4 = have more than two children) and marriage status (1 = single, 2 = married/have a partner)

### Data analysis

We conducted descriptive analysis on demographic data to investigate the characteristics of participants, including their career choice and the places where they currently work. Variability according to gender and preference for PM (first-choice/second-choice) was analyzed with a *χ*^2^ test. Results were considered statistically significant if the two-tailed *p* value was less than .05.

Multiple linear regression analysis was used to describe how multiple life roles and motivational factors were related to the decision to take a PM position and to continue in the PM field. An average score per subscale was calculated; these mean scores per subscale were entered into the regression analysis as variables. Stepwise regression (backward method) was used to test which predictors (i.e., different life roles and motivational factors) predicted the outcome variables (i.e., choosing a PM position or continuing in a PM position). Collinearity diagnostics results showed no multicollinearity between our predictors. The largest variance inflation factor was smaller than 3, and all tolerance values were above .40. All analyses were performed for the whole sample, then separately for men and women and for first-choice and second-choice graduates. *R*^2^ was reported to indicate how much of the variability in the outcome was accounted for by the predictors. Data were analyzed using IBM SPSS Statistics (Version 20.0).

## Results

### General information of participants

Ultimately 167 PM graduates completed the online form (response rate 58%). The mean age of the graduates was 27 years (SD = 2.2; 24 to 38 years), 66.5% were women, and 59.9% chose PM as their first choice when starting medical school. The average number of years since graduation was 4.3 (SD = 1.9); 36.5% still had not decided on career choice. Of all study subjects, 52.7% were working in a PM position, and half of these wanted to stay in the PM field. Participants’ characteristics, timing of career decision, and places of work are described in Table 2.

**Table 2:** Participants’ characteristics, timing of career decision, and places of work

Characteristics of participants	*n*	%
Personal information		
- Married	80	47.9
- Have children	61	36.5
- Born in the countryside	122	73.1
PM preference when starting medical school		
- First choice	100	59.9
- Second choice	64	38.3
- No recall	3	1.8
Places of work		
- Working in PM position	88	52.7
- Non-PM position in private or non-governmental organization	30	18.0
- Clinical doctor in hospital	11	6.6
- Post-graduate student or working in medical school	20	11.9
- Outside health sector	10	5.9
- Looking for employment	8	4.8
Level of public health care organization		
- Central	78	46.6
- Province	57	34.1
- District	32	19.3
- Village	0	0
Timing of decision about career choice		
- Before or at the time of graduation	69	41.3
- During first job	37	22.2
- Had not made a decision	61	36.5
Continuation in PM position		
- Yes	90	53.9
- No	77	46.1

### Association of life roles and motivational factors on graduates’ decision to choose a PM position

Table 3 presents the results on how life’s multiple roles and motivational factors impacted on graduates’ decisions to choose a job in PM for all graduates and subgroups. The results indicated that the “child” role was significantly related to the decision to choose a PM position for all PM graduates (*p* < .01), as was access to continued education (*p* < .001).

**Table 3:** Association between playing multiple life roles and motivational factors on graduates’ decision to choose PM position

	*F*	*df*	*R*^2^	*B*	*SE B*	*β*	95% CIs
All graduates (*N* = 143)	15.5***	*2*	*.2*^*^*^				
Child role				.3	.1	.2**	.1–.6
Continued education				.4	.1	.4***	.2–.6
Male (*N* = 50)	7.9***	3	*.4*^*^*^				
Child role				.7	.2	.5***	.2–.9
Colleagues				.4	.2	.3**	.1–.8
Continued education				.4	.2	.4**	.1–.8
Female *(N* = 93)	11.6***	3	*.3*^^^				
Marital status				.4	.2	.2*	0–.8
Job and working conditions				.4	.1	.3*	.1–.7
Continued education				.4	.1	.3**	.1–.6
First choice (*N* = 82)	12.1***	2	*.2*^*^*^				
Child role				.4	.2	.3**	.1–.7
Continued education				.4	.1	.4***	.2–.6
Second choice (*N* = 58)	5.7*	1	*.1*^*^*^				
Continued education				.4	.2	.3*	.1–.7

Note: *p value ≤ .05, **p value ≤ .01, ***p value ≤ .001, ^^^*p* value > .05; *F* explains the variance in the dependent variable (i.e., decision to choose PM position); *R*^2^ explains how well the model fits the data

Among the subgroups, however, findings were different. Male graduates who valued the child role, satisfaction with colleagues, and satisfaction with continued education were more inclined to choose a PM position. For female graduates, the more they settled down (i.e., married) and the more they were satisfied with their job and working conditions and continued education, the more they tended to choose a PM position. The general model fits well with the first-choice graduates, while the second-choice graduates were more likely to choose a PM job when they were more satisfied with possibilities for continued education.

### Association of life roles and motivation on graduates’ decision to choose a PM position

Table 4 presents the results of how playing multiple life roles and motivational factors impacted graduates’ decision to stay in PM for all graduates and subgroups. The role of “citizen” appeared to be related to continuing in PM among all PM graduates (*p* < .05), as were job and working conditions (*p* < .01) and continued education (*p* < .001).

**Table 4:** Association between playing multiple life roles and motivational factors on graduates’ decision to continue in PM

	*F*	*df*	*R*^2^	*B*	*SE B*	*β*	95% CIs
All graduates (*N* = 131)	8.9***	*3*	*.2*^*^*^				
Citizen role				.5	.2	.2*	.1–.8
Job and working conditions				-.4	.1	-.3**	− .7 to − .1
Continued education				.5	.1	.4***	.3–.8
Male (*N* = 47)	5.8**	2	*.2*^*^*^				
Job and working conditions				-.6	.2	-.5**	− .9 to − .2
Continued education				.6	.2	.5**	.2–.9
Female *(N* = 84)	9.7**	1	*.1*^*^*^				
Continued education				.4	.1	.3**	.1–.7
First choice (*N* = 73)	7.4***	3	*.2*^*^*^				
Citizen role				.7	.3	.5*	.2–1.2
Job and working conditions				-.5	.2	-.4**	− .9 to − .2
Continued education				.6	.2	.5**	.2–.9
Second choice (*N* = 55)	5.9**	2	*.2*^*^*^				
Child role				.4	.2	.3*	0–.8
Continued education				.5	.2	.4**	.2–.8

Note: *p value ≤ .05, **p value ≤ .01, ***p value ≤ .001, ^^^*p* value > .05, *F* explains the variance in the dependent variable (i.e., decision to continue in PM), *R*^2^ explains how well the model fits the data

Findings differed among subgroups. For male graduates, the less they felt satisfied with job and working conditions and the more satisfied with continued education, the more they were inclined to continue in PM. Satisfaction with continued education was the only factor predicting the tendency of staying in PM for female graduates. The general model fits well with the first-choice graduates, while the second-choice graduates would maintain their career in PM if they took the importance of the role of a “child” seriously and were satisfied with possibilities for continued education.

## Discussion

Recruitment and retention of PM staff remains a challenge in many countries. Our study provides new information about PM graduates’ post-educational job choices and interest in continuing to work in this PHC subspecialty, which can inform policy to encourage more PM staff to join and stay in the field. Different factors influenced decisions on job choice and continuing in PM among men and women and between graduates who had or had not chosen PM at the start of their university study.

The results showed that only half of PM graduates were actually working in PM, the majority at the central and provincial levels, while about 10% of half of the graduates worked in hospitals or outside the health sector, and none of them worked in the community. This is consistent with existing evidence that PHC staff in Vietnam move away from the community and from preventive care [12, 14]. In the current study, nearly 40% of the graduates had chosen to study PM without a strong preference, that is, as a second choice. As we have previously reported [11], PM students had misconceptions about what PM doctors really do in practice, which might partly explain graduates’ job choices, although we did not find a significant difference in current jobs between graduates who chose PM as a first or second choice.

In this study, we found that one third of graduates had not decided which specialty to pursue as a career, even though half of them had graduated more than 3 years earlier, and one third were already working in PM. This career decision-making time period is longer than that from previous findings among medical graduates [18, 19] or among more mature graduates of clinical specialties such as nephrology [27] or oncology [28], which reported the time was 1 to 3 years after qualification. This indecision about career choice among graduates in a PHC specialty has been confirmed in both developed [18, 19] and developing countries [10, 13]. The possibility of losing more than half of the graduates who did not choose PM even 3 years after qualification questions the effectiveness of training programs that recruit freshmen straight to the PHC subspecialty training, suggesting that the specialty should be offered as a post-graduate training specialty. However, to be more optimistic, this 3-year period of instability could be seen as an opportunity to engage more graduates who are still undecided, by working to meet their needs and nurturing their preference for work in PHC.

Concerning the influence of life roles on graduates’ career decisions, we found a significant association between the “role of a child” (i.e., staying close to, respecting, and taking care of parents) and the decision to choose a PM job. Additionally, the “role of a citizen” (i.e., serving the community) was related to the decision to stay in the PM field, although not in all subgroups. Responsibility to parents only influenced the choice for PM among male and first-choice graduates. This confirms an observation in our previous study [11], which showed that first-choice PM students were drawn to study PM by the desire to uphold their family traditions and fulfill their parents’ wishes. The fact that the role of a child strongly affected male graduates but not their female peers could be explained by the social norm in Vietnam that expects men to take care of their parents, while women should assist their husbands in that task. Interestingly, a child’s responsibility for their parents encourages particularly second-choice graduates to stay in PM, even though it might not be their preferred specialty. The idea of working in PM, with its offer of stability and sufficient time to care for parents, might help to explain this motivation. This interpretation is supported by another report [14] that Vietnamese doctors expressing interest in stable work and salary were more satisfied with their current job in rural areas. For female graduates, marriage was a significant factor influencing the choice of a PM job, consistent with previous findings in European countries [17, 19]. These advantages of PM work could be emphasized to increase the attractiveness of the specialty to new students and undecided young doctors, especially to women and second-choice graduates.

Interestingly, we found a link between commitment to serve the community (the citizen role) and the tendency to stay in PM, particularly among those for whom PM was their first choice. This confirms the importance of intrinsic factors, such as “being responsible” and “relation with the community” that have been noted as significant motivators from the perspective of health professionals in developing countries [16, 17]. Although our data do not allow us to conclude that the inherent preference for PM of first-choice graduates was the main motivator for them to stay in the field, it might be useful to guide recruitment of “the right students” and “the right staff” in a less popular specialty like PHC. Additionally, our findings enrich the existing application of Super’s role salience theory in human resources development [22] by providing information from the perspective of both health care-related human resources and that of a developing Asian country whose values and cultural context are different from the Western settings the theory and its related literature have mostly focused on. However, we did not investigate the interaction among the roles important to health workers during their career development, including the influence of changing life roles on an individual’s career development strategies. Future longitudinal studies can help in the understanding of the inter-role relationships and the effect of role fluctuation in different life stages on retention of PHC health workers.

Along with the impact of personal life roles, we found that satisfaction with opportunities for continued education was prominent in graduates’ motivation, regardless of gender or PM preference. This motivating factor can be linked to the highest level of Maslow’s pyramid, that is self-actualization, as access to continued education would provide graduates opportunities for professional achievement and advancement. This result is in line with a previous study on motivation of medical staff in a Cyprus public general hospital [29]; however, our results did not reveal a connection of certain factors including remuneration or good relationships with coworkers to lower levels of the pyramid, as reported in that study. Critics of Maslow’s hierarchy of needs also assert that this theory may not be universally applicable and may vary across cultural, organizational, and individual perspectives in different countries [30]. Another possible explanation for this difference is that our participants are PHC workers, a specialty that is considered to have less prestige and be less attractive than hospital-based specialties. Therefore, their desire might be for the opportunity for achievement and to gain respect from colleagues and the community, which can be linked to the achievement needs in Maslow’s pyramid. Our findings also confirm the observation of Witter et al. [14], who listed the lack of opportunity for further training or continued education as an impediment to working in PHC. Interestingly, these authors suggested that a possible underlying reason for health workers’ demands for further training was that better training might be a “passport” for them to leave their lower-level facilities. An implication of our findings on retaining PM doctors therefore includes both providing more trainings (short courses, graduate, and post-graduate) and controlling the final destination of PM staff after achieving higher qualifications. Unexpectedly, we found a negative relationship between satisfaction with job and working conditions and the decision to stay in PM, especially among men and first-choice graduates who decided to stay because of their responsibility to their parents. Our data do not explain this unusual connection; we hypothesize that these graduates felt unhappy because they did not see any other option than continuing in PM. Further study is necessary to explore this issue in more detail.

## Limitations

The present study has several limitations. Firstly, the study design was cross-sectional, which allowed us to examine predictors’ association with PM recruitment and retention at only a single time point. In the future, longitudinal studies should be conducted to follow participants and describe the entire process of their career choices, from the beginning to the final decision. Secondly, participants were PM graduates drawn from one medical school; when this study was conducted, six out of 13 Vietnamese state medical universities offered PM training, including HMU. To increase the generalizability of our results, future studies should recruit graduates from other medical schools that also provide PM training, as differences in training programs, sociodemographic characteristics, facilities, and available positions might impact job choices. Finally, the limited sample size was partly a result of the university’s not yet well-established alumni information system, which caused random missing values. The relatively low response rate from graduates whom we did reach might not be fully representative of our sample, for example, alumni who are dissatisfied with their current situation could have been more inclined to answer than those who are satisfied. Future study could also include the 4-year program graduates, whose motivations may be different because they already have practical health care experience before starting their study. Their perspectives would be relevant and might provide good information to policy makers to focus efforts on people with more experience.

## Conclusions

This study, which asked graduates from a PHC subspecialty training program what motivated them to choose a PHC job and to keep working in the field, is the first of its kind. The research provided information about timing of career choices, personal needs, and expectations of graduates that will support the design of interventions aimed at maintaining an adequate PHC workforce. The findings highlight the importance of continuing education and the role of graduates in relation to family and the community in recruiting and retaining health workers in PHC in Vietnam. These findings can inform and inspire policy development to manage human resources for PHC in Vietnam and perhaps globally and might also be useful for other less popular specialties struggling with recruiting and retaining staff.

## Supplementary information

**Additional file 1.** Survey questionnaire of PM graduates

## Data Availability

The datasets generated and analyzed during the current study are available in the Maastricht University Dataverses/School of Health Professions Education (SHE) [31].

## Abbreviations

HMU: Hanoi Medical University
MLICs: Middle- and low-income countries
PHC: Primary health care
PM: Preventive medicine
