# Supplementary material for: Why do graduates choose to work in a less attractive specialty? A cross-sectional study on the role of personal values and expectations
Source: Hum Resour Health. 2020 May 4;18:32. doi: 10.1186/s12960-020-00474-y (PMC7197171; doi:10.1186/s12960-020-00474-y)
Supplement: Supplementary file 1 — Additional file 1. Survey questionnaire of PM graduates [file 12960_2020_474_MOESM1_ESM.docx]

Appendix 1: Survey questionnaire of PM Graduates

| **No** | **Question** | **Answer** | | | | | | |  | |
| --- | --- | --- | --- | --- | --- | --- | --- | --- | --- | --- |
| **A** | **Personal information** | ***Tick the corresponding box*** | | | | | | |  | |
| A1 | How old are you? | ……. old | | | | | | |  | |
| A2 | What is your gender? | Male  Female | | | | | | | 1🗖  2🗖 | |
| A3 | What is your marital status? | Single or divorced  Married  Others (in detail): …… | | | | | | | 1🗖  2🗖  97🗖 | |
| A4 | How many children do you have? | …… | | | | | | |  | |
| A5 | Which was the level of priority of your choice at medical school to study PM? | PM was my first preference  PM was my second preference  PM was my third preference  I do not remember | | | | | | | 1🗖  2🗖  3🗖  98🗖 | |
| A6 | Where are you working? | State health establishment  Private health center  Foreign organization  Medical school/institute/college  Post graduate studying  Clinical department in hospital  Para-clinical department  Not working in medicine  Unemployment  Others (in detail): ……………………… | | | | | | | 1🗖  2🗖  3🗖  4🗖  5🗖  6🗖  7🗖  8🗖  9🗖  97🗖 | |
| A7 | If you are working in a state health establishment, which level are you working at? | Central  Provincial  District  Commune  Lecturer/researcher  Others (in detail): …… | | | | | | | 1🗖  2🗖  3🗖  4🗖  5🗖  97 🗖 | |
| A8 | When did you make a final decision on the specialty in which you want to pursue your career? | When I chose to study PM  When I was studying at medical school  When I finished studying and graduated  When I found my first job  When I found my current job  I haven’t made the decision yet | | | | | | | 1🗖  2🗖  3🗖  4🗖  5🗖  6🗖 | |
| **B** | **Job Choice and Retention in PM field** | **Please mark on the scale to which you agree or disagree about your current job (1-Strongly disagree; 2-Partly disagree; 3-Neither agree nor disagree; 4-Partly agree; 5-Strongly agree; 6-Not applicable)** | | | | | | | | |
|  |  | (1) | (2) | (3) | | (4) | | (5) | | (6) |
|  | ***Decision on taking a job offer*** |  |  |  | |  | |  | |  |
| B1 | I accepted this job because it was the only available job offer I had at that time | 🗖 | 🗖 | 🗖 | | 🗖 | | 🗖 | |  |
| B2 | I accepted this job because it helps me to have stable incomes | 🗖 | 🗖 | 🗖 | | 🗖 | | 🗖 | |  |
| B3 | I had tried several jobs and was not happy until this one | 🗖 | 🗖 | 🗖 | | 🗖 | | 🗖 | |  |
| B4 | I accepted this job because it seemed to be the best offer among several job options I had | 🗖 | 🗖 | 🗖 | | 🗖 | | 🗖 | |  |
| B5 | I accepted this job because it fits best with my expectations and desires about a job | 🗖 | 🗖 | 🗖 | | 🗖 | | 🗖 | |  |
| B6 | I accepted this job because it relates the most to PM field | 🗖 | 🗖 | 🗖 | | 🗖 | | 🗖 | | 🗖 |
| B7 | I gathered as much information as possible about the job before I accepted it | 🗖 | 🗖 | 🗖 | | 🗖 | | 🗖 | |  |
| B8 | My current job is well fit with what I had been trained for | 🗖 | 🗖 | 🗖 | | 🗖 | | 🗖 | |  |
|  | ***Retention in PM field*** |  |  |  | |  | |  | |  |
| B9 | I will probably look for a new job in the upcoming year | 🗖 | 🗖 | 🗖 | | 🗖 | | 🗖 | |  |
| B10 | I often think about quitting this job | 🗖 | 🗖 | 🗖 | | 🗖 | | 🗖 | |  |
| B11 | I wish to work in PM field if I have the opportunity | 🗖 | 🗖 | 🗖 | | 🗖 | | 🗖 | | 🗖 |
| B12 | I wish to leave PM field if I can find another job | 🗖 | 🗖 | 🗖 | | 🗖 | | 🗖 | | 🗖 |
| B13 | I think I will keep this job as long as possible | 🗖 | 🗖 | 🗖 | | 🗖 | | 🗖 | |  |
| **C** | **Job satisfaction** | **Please mark on the scale to which you agree or disagree about your current job (1-Strongly disagree; 2-Partly disagree; 3-Neither agree nor disagree; 4-Partly agree; 5-Strongly agree; 6-Not applicable)** | | | | | | | | |
|  |  | (1) | (2) | (3) | (4) | | (5) | | | (6) |
|  | ***Satisfaction on “Job and working condition”*** | | | | | | | | | |
| C1 | I like doing the things I do at work | 🗖 | 🗖 | 🗖 | 🗖 | | 🗖 | | |  |
| C2 | I sometimes feel my job is meaningless | 🗖 | 🗖 | 🗖 | 🗖 | | 🗖 | | |  |
| C3 | My job is enjoyable | 🗖 | 🗖 | 🗖 | 🗖 | | 🗖 | | |  |
| C4 | I am satisfied with my job in terms of working conditions | 🗖 | 🗖 | 🗖 | 🗖 | | 🗖 | | |  |
| C5 | I feel a sense of pride in doing my job | 🗖 | 🗖 | 🗖 | 🗖 | | 🗖 | | |  |
|  | ***Satisfaction on “Management skills”*** | | | | | | | | | |
| C6 | My manager provides me assistance at difficult cases | 🗖 | 🗖 | 🗖 | 🗖 | | 🗖 | | |  |
| C7 | My manager is quite competent in doing his/her job | 🗖 | 🗖 | 🗖 | 🗖 | | 🗖 | | |  |
| C8 | My manager manages his subordinates well | 🗖 | 🗖 | 🗖 | 🗖 | | 🗖 | | |  |
| C9 | My manager always stands behind the workers | 🗖 | 🗖 | 🗖 | 🗖 | | 🗖 | | |  |
| C10 | My manager considers the complaints of employees | 🗖 | 🗖 | 🗖 | 🗖 | | 🗖 | | |  |
|  | ***Satisfaction on “Co-workers”*** | | | | | | | | | |
| C11 | I am satisfied with my colleagues on being agreeable with each other | 🗖 | 🗖 | 🗖 | 🗖 | | 🗖 | | |  |
| C12 | I am satisfied with the people I speak and work with | 🗖 | 🗖 | 🗖 | 🗖 | | 🗖 | | |  |
| C13 | My colleagues are hardworking | 🗖 | 🗖 | 🗖 | 🗖 | | 🗖 | | |  |
| C14 | I am satisfied with the friendship of my colleagues | 🗖 | 🗖 | 🗖 | 🗖 | | 🗖 | | |  |
| C15 | My colleagues take responsibility for their duties | 🗖 | 🗖 | 🗖 | 🗖 | | 🗖 | | |  |
|  | ***Satisfaction on “Promotion”*** | | | | | | | | | |
| C16 | I am satisfied with the opportunity of being promoted at this job | 🗖 | 🗖 | 🗖 | 🗖 | | 🗖 | | |  |
| C17 | Promotion at my work is based on individual skills | 🗖 | 🗖 | 🗖 | 🗖 | | 🗖 | | |  |
| C18 | The person who performs well in his job can get the chance to be promoted | 🗖 | 🗖 | 🗖 | 🗖 | | 🗖 | | |  |
| C19 | There are regular promotion practices at my work | 🗖 | 🗖 | 🗖 | 🗖 | | 🗖 | | |  |
|  | ***Satisfaction on “Pay”*** | | | | | | | | | |
| C20 | My wage is enough for my regular expenses | 🗖 | 🗖 | 🗖 | 🗖 | | 🗖 | | |  |
| C21 | I feel satisfied with my salary increases | 🗖 | 🗖 | 🗖 | 🗖 | | 🗖 | | |  |
| C22 | My wage is appropriate when it is compared with workers in other occupations | 🗖 | 🗖 | 🗖 | 🗖 | | 🗖 | | |  |
| C23 | I think I get a fair wage for the work I do | 🗖 | 🗖 | 🗖 | 🗖 | | 🗖 | | |  |
| C24 | My salary is good when it is compared with the wage of other physicians who work at similar positions in other specialties | 🗖 | 🗖 | 🗖 | 🗖 | | 🗖 | | |  |
|  | ***Satisfaction on “Contingent rewards”*** | | | | | | | | | |
| C25 | I do not feel that the work I do is appreciated | 🗖 | 🗖 | 🗖 | 🗖 | | 🗖 | | |  |
| C26 | I don't feel my efforts are rewarded the way they should be | 🗖 | 🗖 | 🗖 | 🗖 | | 🗖 | | |  |
| C27 | When I do a good job, I receive the recognition for it that I should receive | 🗖 | 🗖 | 🗖 | 🗖 | | 🗖 | | |  |
| C28 | I feel respected and supported while working with people in the community | 🗖 | 🗖 | 🗖 | 🗖 | | 🗖 | | | 🗖 |
| C29 | I feel respected and supported by medical colleagues in other specialties | 🗖 | 🗖 | 🗖 | 🗖 | | 🗖 | | |  |
| C30 | There are few rewards for those who work in my organization | 🗖 | 🗖 | 🗖 | 🗖 | | 🗖 | | |  |
|  | ***Satisfaction on “Continued education”*** | | | | | | | | | |
| C31 | I feel satisfied with my possibilities for continued education | 🗖 | 🗖 | 🗖 | 🗖 | | 🗖 | | |  |
| C32 | I have gained a lot of PM practical knowledge and skills from doing this job | 🗖 | 🗖 | 🗖 | 🗖 | | 🗖 | | |  |
| C33 | My opportunities for continued education are appropriate when compared to physicians who work in other specialties | 🗖 | 🗖 | 🗖 | 🗖 | | 🗖 | | |  |
| C34 | I am supported by managers and colleagues while I have to study for my continued education | 🗖 | 🗖 | 🗖 | 🗖 | | 🗖 | | | 🗖 |
| C35 | Chances for continued education at my work are based on individual competence and needs | 🗖 | 🗖 | 🗖 | 🗖 | | 🗖 | | |  |
| ***D*** | ***Roles in life*** | **Please mark on the scale to which you agree or disagree about your roles in life (1-Strongly disagree; 2-Partly disagree; 3-Neither agree nor disagree; 4-Partly agree; 5-Strongly agree; 6-Not applicable)** | | | | | | | | |
|  |  | (1) | (2) | (3) | (4) | | (5) | | | (6) |
|  | ***Child role*** | | | | | | | | | |
| D1 | I tried to find a job with which I can afford to support my parents and family in my hometown | 🗖 | 🗖 | 🗖 | 🗖 | | 🗖 | | | 🗖 |
| D2 | I accepted a job offer because my parents wanted me to do so | 🗖 | 🗖 | 🗖 | 🗖 | | 🗖 | | | 🗖 |
| D3 | I tried to find a job which allowed me to have time for my parents | 🗖 | 🗖 | 🗖 | 🗖 | | 🗖 | | | 🗖 |
| D4 | Although my parents might express their wishes, I do have my own decision when considering to accept a job offer | 🗖 | 🗖 | 🗖 | 🗖 | | 🗖 | | | 🗖 |
| D5 | I tried to find a job which was close to my parents’ house | 🗖 | 🗖 | 🗖 | 🗖 | | 🗖 | | | 🗖 |
|  | ***Parental role*** | | | | | | | | | |
| D6 | I tried to find a job with which I can afford to send my children to good schools | 🗖 | 🗖 | 🗖 | 🗖 | | 🗖 | | | 🗖 |
| D7 | I tried to find a job which allowed me to have time to take care of my children | 🗖 | 🗖 | 🗖 | 🗖 | | 🗖 | | | 🗖 |
| D8 | It is important to me to have some time for myself and my own development rather than have children and be responsible for their care | 🗖 | 🗖 | 🗖 | 🗖 | | 🗖 | | | 🗖 |
| D9 | Becoming involved in the day-to-day details of rearing children involves costs in other areas of my life which I am unwilling to make | 🗖 | 🗖 | 🗖 | 🗖 | | 🗖 | | | 🗖 |
| D10 | I am willing to devote a significant amount of my time and energy to the rearing of my children | 🗖 | 🗖 | 🗖 | 🗖 | | 🗖 | | | 🗖 |
| D11 | I am not willing to be very involved in child rearing | 🗖 | 🗖 | 🗖 | 🗖 | | 🗖 | | | 🗖 |
|  | ***Marital role*** | | | | | | | | | |
| D12 | I tried to find a job which was close to the place where my partner was working | 🗖 | 🗖 | 🗖 | 🗖 | | 🗖 | | | 🗖 |
| D13 | I tried to find a job which allowed me to have time for my marriage partner | 🗖 | 🗖 | 🗖 | 🗖 | | 🗖 | | | 🗖 |
| D14 | I am willing to put a lot of time and effort into building and maintaining a marriage relationship | 🗖 | 🗖 | 🗖 | 🗖 | | 🗖 | | | 🗖 |
| D15 | I am willing to work hard to build a good marriage relationship even if it means limiting my opportunities to pursue other personal goals | 🗖 | 🗖 | 🗖 | 🗖 | | 🗖 | | | 🗖 |
| D16 | Really involving myself in a marriage relationship involves costs in other areas of my life which I am unwilling to accept | 🗖 | 🗖 | 🗖 | 🗖 | | 🗖 | | | 🗖 |
| D17 | Devoting a significant amount of my time to being with or doing things with a marriage partner is not something I want to do | 🗖 | 🗖 | 🗖 | 🗖 | | 🗖 | | | 🗖 |
|  | ***Occupational role*** | | | | | | | | | |
| D18 | I tried to find a job that was interesting and exciting to me | 🗖 | 🗖 | 🗖 | 🗖 | | 🗖 | | |  |
| D19 | I am willing to make as many sacrifices as necessary in order to advance in my work/career | 🗖 | 🗖 | 🗖 | 🗖 | | 🗖 | | |  |
| D20 | I want to work, but I do not want to have a demanding career | 🗖 | 🗖 | 🗖 | 🗖 | | 🗖 | | |  |
| D21 | I am willing to devote whatever time and energy it takes to move up in my job/career field | 🗖 | 🗖 | 🗖 | 🗖 | | 🗖 | | |  |
| D22 | I value being involved in a career and want to devote the time and effort needed to develop it | 🗖 | 🗖 | 🗖 | 🗖 | | 🗖 | | |  |
| D23 | I am willing to devote a significant amount of my time to building my career and developing the skills necessary to advance in my career | 🗖 | 🗖 | 🗖 | 🗖 | | 🗖 | | |  |
|  | ***Homecare role*** | | | | | | | | | |
| D24 | I want to have the responsibility for seeing that my home is well kept and well run | 🗖 | 🗖 | 🗖 | 🗖 | | 🗖 | | |  |
| D25 | I tried to find a job which allowed me to have time to manage and care for my home | 🗖 | 🗖 | 🗖 | 🗖 | | 🗖 | | |  |
| D26 | I tried to find a job which was near my house | 🗖 | 🗖 | 🗖 | 🗖 | | 🗖 | | |  |
| D27 | I am not willing to devote a significant amount of my time to managing and caring for a home | 🗖 | 🗖 | 🗖 | 🗖 | | 🗖 | | |  |
| D28 | I want to leave most of the day-to-day details of running a home to someone else | 🗖 | 🗖 | 🗖 | 🗖 | | 🗖 | | |  |
| D29 | I want to be very much involved in caring for a home and making it attractive | 🗖 | 🗖 | 🗖 | 🗖 | | 🗖 | | |  |
|  | ***Citizen role*** | | | | | | | | | |
| D30 | I tried to find a job in which I can contribute to the community where I originate from | 🗖 | 🗖 | 🗖 | 🗖 | | 🗖 | | |  |
| D31 | I tried to find a job that could help me to stay in big cities | 🗖 | 🗖 | 🗖 | 🗖 | | 🗖 | | |  |
| D32 | I am willing to devote the time and effort needed to develop my original community | 🗖 | 🗖 | 🗖 | 🗖 | | 🗖 | | |  |
| D33 | At this moment having a successful career and family life is more important than caring for my own community | 🗖 | 🗖 | 🗖 | 🗖 | | 🗖 | | |  |

**Thank you very much for your time!**
